# Application of Augmented Reality Navigation in Craniofacial Surgery for Fibrous Dysplasia

**DOI:** 10.1055/a-2547-5400

**Published:** 2025-04-11

**Authors:** Will C. Kaiser, Sanaa Hameed, Fauziyya Muhammad, David Barkyoumb, Christian El Amm, Zachary A. Smith

**Affiliations:** 1 Department of Neurosurgery, University of Oklahoma Health Sciences Center, Oklahoma City, Oklahoma, United States; 2 Section of Plastic Surgery, Department of Surgery, University of Oklahoma Health Sciences Center, Oklahoma City, Oklahoma, United States

**Keywords:** augmented reality, craniofacial, fibrous dysplasia, surgical navigation

## Abstract

**Introduction:**

Fibrous dysplasia of the craniofacial bones, or craniofacial dysplasia (CFD), involves the replacement of normal bone with fibrous osseous tissue, resulting in asymmetry and distortion of the overlying soft tissue and irregular bone deposition. Treatment primarily involves surgical resection, and achieving symmetry by matching the contralateral unaffected side is crucial. However, surgical correction is challenging due to the lack of visualization of the normal contralateral structures and the need to precisely control resection depth. Although the application of an augmented reality navigation (ARN) system for CFD surgery has been documented, to our knowledge its specific use in identifying key neurovascular structures has not been reported.

**Methods:**

We present the application of an ARN system for the surgical management of an 18-year-old woman with CFD. The virtual plan was designed to visualize the extent of tumor, identify normal and abnormal vasculature, and guide the reconstruction of a normal anatomical contour.

**Results:**

ARN was successfully integrated into the surgical workflow and optimized operative planning, identification of tumor margins, avoidance of neurovascular structures, reconstruction, and symmetric recontouring. The ability to visualize structures in real time proved to be especially beneficial for making intraoperative adjustments.

**Conclusion:**

ARN has significant applications for CFD surgery by providing real-time, three-dimensional simulation, and precise overlay of patient-specific anatomy and pathology, facilitating safe resection, and providing a useful reconstruction guide. To our knowledge, this report presents the first detailed description of its utility in visualizing critical neurovascular structures, offering significant potential to enhance surgical safety and patient outcomes.

## Introduction

Fibrous dysplasia is a benign hamartomatous bone disease characterized by the proliferation and abnormal deposition of fibrous tissue, causing deformities and functional impairments. 1 Fibrous dysplasia involving the craniofacial bones, or craniofacial dysplasia (CFD), typically presents as a painless, expansive mass leading to deformity and asymmetry of the craniofacial skeleton. 2 3 Complications include hearing and vision impairment, nasal obstruction, sinusitis, headaches, discomfort, aesthetic impairment, and malocclusion. 3 The primary treatment is surgical resection, which involves corrective osteotomy, debulking, and recontouring to achieve optimal facial symmetry and restore function. 2 4 However, the facial region's complex anatomy and proximity to critical neurovascular structures creates challenges unique to CFD surgery. 5

Augmented reality navigation (ARN) technology integrates preoperative planning and intraoperative guidance by generating real-time, three-dimensional (3D) virtual models of patient-specific anatomy, which is then superimposed onto the operative field. This enhances spatial orientation and depth perception, allowing simultaneous visualization of the normal contralateral anatomy. 6 7 This is compared with traditional freehand techniques, which may be limited by factors such as obscuration of anatomical landmarks, making visual estimation challenging and potentially leading to suboptimal contouring. 8 Such capabilities improve surgical precision, especially in craniofacial procedures, where the accurate restoration of functional anatomy is key. 9

Here, we present the novel application of an advanced ARN-assisted system in the recontouring surgery of an 18-year-old CFD patient. Although several reports have described the utilization of ARN in CFD surgery, 10 11 this report highlights the role of ARN in visualizing the aberrant hypervascular patterns that complicate surgery of fibrous dysplasia in its active phase.

## Illustrative Case

**Fig. 1:**
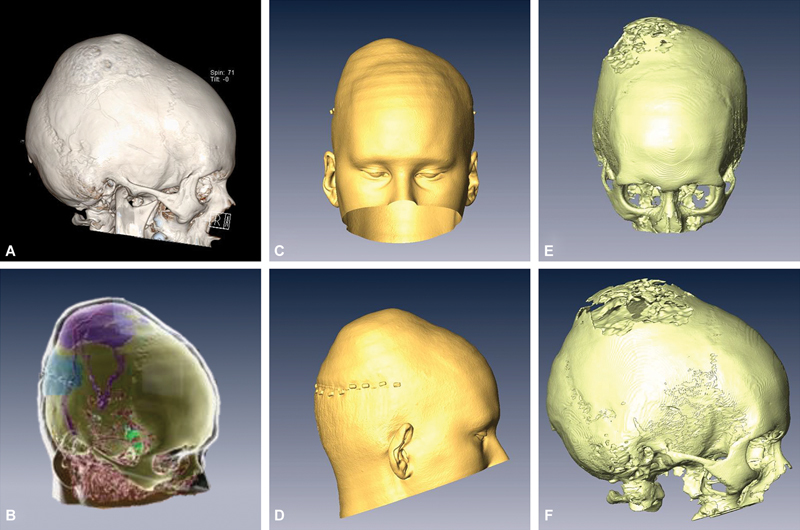
Preoperative imaging and 3D ARN model creation. CT with 3D reconstruction demonstrates fibrous dysplasia of the superior aspect of the right parietal bone ( **A** ). These reconstructions were then uploaded into the IntraOpVSP software to create the 3D model (B), including both skin ( **C, D** ) and bone ( **E, F** ) renderings that are utilized by the ARN system intraoperatively. 3D, three-dimensional; ARN, augmented reality navigation; CT, computed tomography.

An 18-year-old female with an 8-year history of fibrous dysplasia presented with significant bony overgrowth of the right parietal bone, progressively worsening headaches, visual changes, photo- and phonophobia with nausea, and dysmenorrhea that had been developing over the last 5 years. Blood testing for McCune–Albright syndrome was negative. She also suffered severe migraines unresponsive to botulinum toxin injections. Visual acuity had been evaluated as fibrous dysplasia that involved the right orbital roof. She had also complained of neuropathic pain with tingling and numbness in the regions over the temporal crest bilaterally with secondary tightness of her sternocleidomastoid muscle. The remaining neurological exam appeared normal. On recent examination, there was an increase in size of the right parietal tumor with diffuse tenderness of four trigger points. Computed tomography with 3D reconstruction revealed a right posterior parietal bone prominence measuring 12 cm × 7 cm × 4 cm indicative of CFD ( Fig. 1A ). Given the increasing size of the tumor with associated tenderness, the decision was made to proceed with excision of the tumor and reconstruction.

### Surgical Planning with Augmented Reality System

**Fig. 2:**
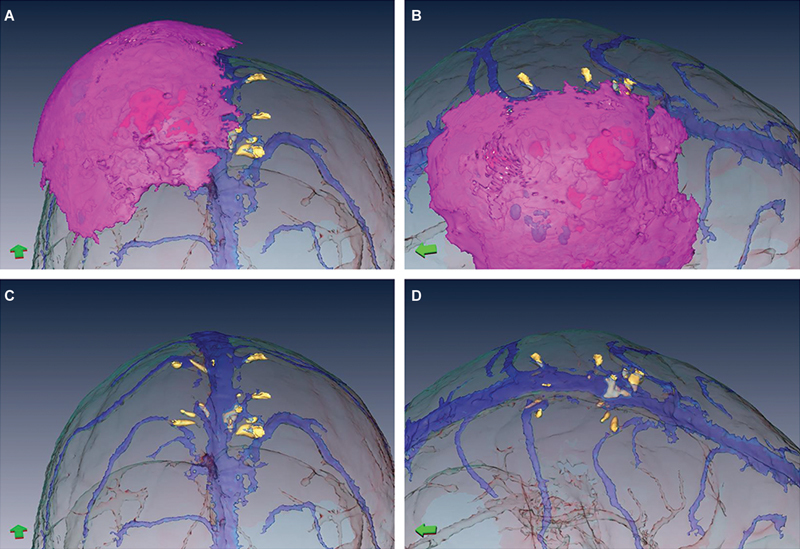
Preoperative 3D model constructed within the IntraOpVSP software depicting the bone tumor (pink mass), superior sagittal sinus and surrounding veins (dark blue structures), and bone spicules (yellow consolidations) via frontal ( **A** ) and vertex ( **B** ) orientations. Panels ( **C** ) and ( **D** ) represent the model with the tumor reconstruction lifted, thus allowing more detailed visualization of the venous structures and bone spicules.

Contrast-enhanced computed tomography (CT) and magnetic resonance imaging (MRI) data are segmented according to established methods to generate patient-specific 3D models of soft tissues, osseous anatomy, tumor, and adjacent vasculature. 12 The 3D model developed for the patient can be seen in Figs. 1 and 2 . The abnormal contour is delineated, and the corrected contour is generated using mirror-image templates and finalized within the IntraOpVSP system (Xironetic, LLC, Oklahoma City, Oklahoma, United States). An extended reality head-mounted VR headset display was worn by the operating physician and assistants. The IntraOpVSP system is then overlayed through the head-mounted display and allows for control of orientation, cross-sections, and removal of the layered visualization of the anatomy in addition to segmentation of areas of interest.

### Surgical Procedure

**Fig. 3:**
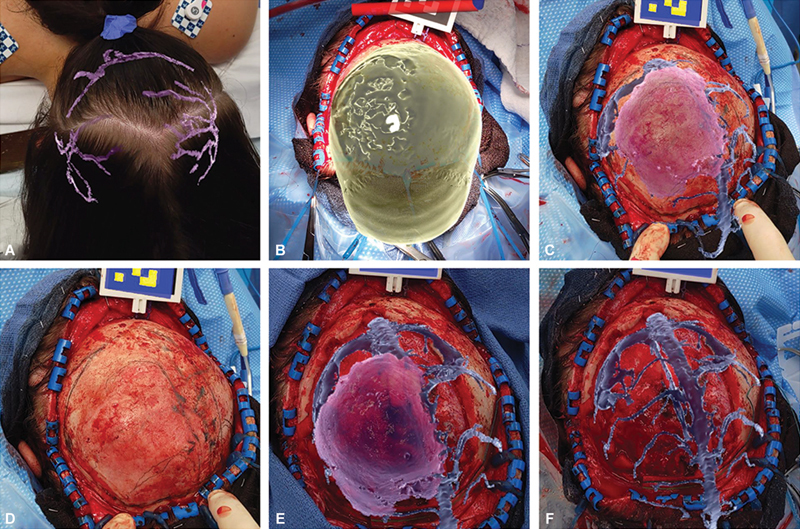
Intraoperative images demonstrating the use of the ARN technology at different points of the procedure. Planning of the skin incisions was guided by overlay of the large scalp veins ( **A** ). Following flap elevation, the bone model was overlayed, thus confirming proper orientation ( **B** ). Tumor and sinus anatomy, along with the bone spicules, were then overlayed ( **C** ), clearly mapped, and marked with pencil ( **D** ), facilitating the planning of the craniectomy. These structures were also overlayed during the procedure and provided real-time feedback, ensuring the boundaries of the sinus and tumor were respected during the bone resection. Following craniectomy, the tumor, sinus and dural vessels demonstrated excellent correspondence with the ARN overlay ( **E, F** ). ARN, augmented reality navigation.

**Fig. 4:**
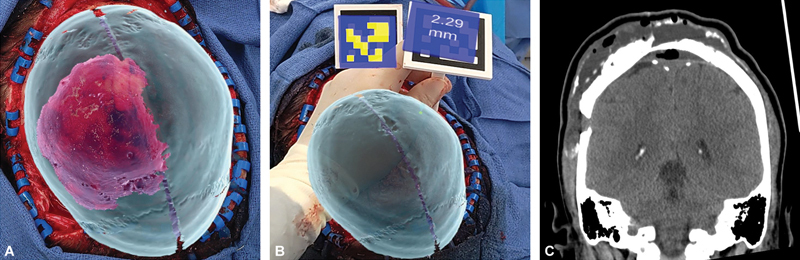
Utilization of ARN overlay to guide contouring of the affected region based on the contralateral unaffected anatomy, thus ensuring symmetric reconstruction ( **A** ). The ARN stylus was used to measure the distance between the current and planned bone surface, allowing for real-time adjustments during surgery ( **B** ). Postoperatively, the patient experienced a small residual seroma over the right vertex that required evacuation and drainage ( **C** ). ARN, augmented reality navigation.

Utilization of the ARN system throughout various points of the procedure is depicted in Figs. 3 and 4 . With the patient in the prone position, the extent of the tumor was exposed via a subperiosteal approach and additional margins dissected based on the ARN depiction of the intraosseous extent of the tumor and feeding blood vessels. ARN was then utilized to identify the margins of the fibrous dysplasia over the right parietal skull. Sinuses and large branching veins were defined with the assistance of the neurosurgery team. The bone flap, including the tumor, was removed and the accuracy of the virtual anatomy verified. Because ARN allowed for easy visualization of the sagittal sinus and surrounding vasculature, we were able to promptly control the two major feeding emissary veins, as well as additional lesser perforating veins, thus limiting blood loss. The bone flap and tumor were then contoured ex vivo using the bone graft harvested in situ. The reconstructed bone flap was secured to the unaffected calvarium, the contour verified using the ARN distance measurement tool, and visualization of the reference contour mirrored from the unaffected contralateral anatomy ( Fig. 4B ).

### Postoperative Course

**Fig. 5:**
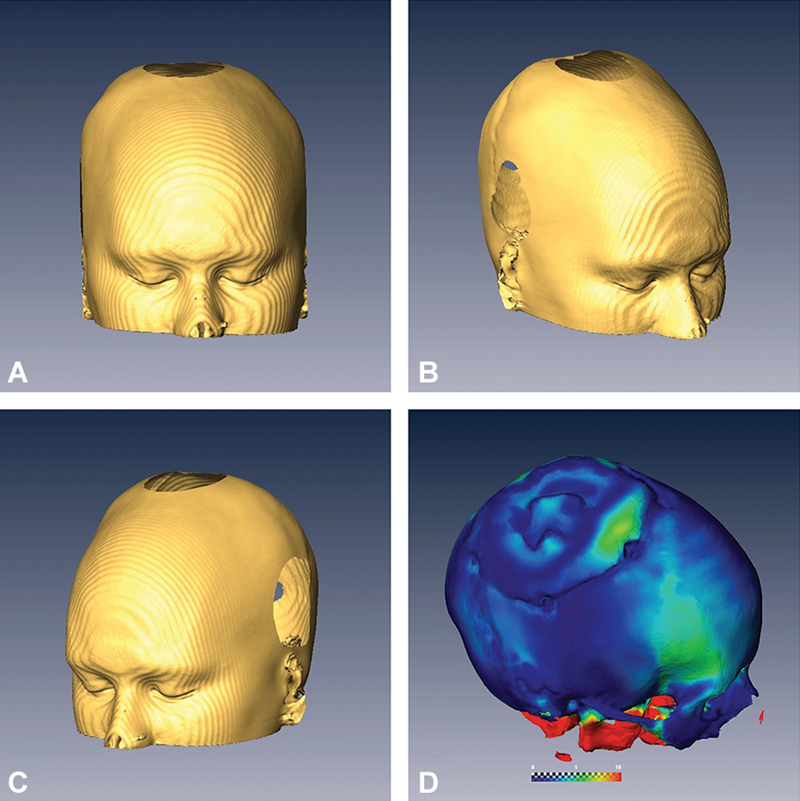
Postoperative 3D reconstructions confirming successful removal of the CFD and restoration of normal cranial contours ( **A** – **C** ). Quantitative comparison between preoperative and postoperative bone surfaces indicated that 85% of the tumor resection falls within 2 mm of the planned contour, whereas 94% of the tumor surface is within 5 mm of the desired contour, demonstrating a high level of surgical accuracy ( **D** ). 3D, three-dimensional; CFD, craniofacial dysplasia.

The postoperative period was uneventful except for a small residual seroma over the right vertex that required evacuation and drainage ( Fig. 4C ). Postoperative 3D reconstructions confirmed successful removal of the tumor and restoration of normal cranial contours ( Fig. 5 ). At 6-month follow-up, the patient demonstrated markedly improved contour with only one residual trigger point at a site of protrusion in the right temporal region.

## Discussion

In this case, we utilized ARN to assist in preoperative planning and intraoperative guidance for the resection of posterior parietal dysplastic bone in an 18-year-old CFD patient. ARN enhanced synchronization between preoperative imaging and intraoperative navigation, helping us overcome limitations in freehand techniques. It allowed real-time visualization of 3D anatomy, lesion mapping, and refinement of the preoperative plan, thus improving accuracy by enabling visualization of dysplastic bone and critical neurovascular structures, particularly the dural vessels and their anatomical relationship to the tumor. This significantly improved the safety of the surgery by reducing the likelihood of inadvertent damage to these structures, which is typically very challenging without ARN due to disrupted anatomy. ARN was also beneficial as a contouring guide, in which the mirror image of the unaffected side was used as a reference.

ARN offers comprehensive utility throughout all phases of surgical CFD management. Preoperatively, a 3D virtual model overlay onto the skull allows manipulation of specific structures via voice commands to simulate each surgical step, plan optimal incision placement, and anticipate challenges. Intraoperatively, ARN enables real-time visualization of critical structures, such as the tumor and sagittal sinus, which are more precise compared with traditional imaging techniques like MRI or CT. 13 This feature also reduces operating time. 14 15 Additionally, ARN helped ensure cranial symmetry by providing real-time feedback on the distance between the dysplastic bone and the planned contour, which was facilitated by a mirror image of the unaffected side, allowing for immediate adjustments.

Previous literature, including reports by Gao et al 10 and Liu et al, 11 has documented the use of ARN in surgical recontouring for CFD, primarily focusing on intraoperative visualization of patient anatomy and lesion extent. However, these studies have not fully explored the registration of models, with the purpose of visualizing the precise positions of critical structures such as blood vessels during the procedure. The ability to visualize these structures and their relationship to the dysplastic bone in real time greatly enhances both the safety of the surgery and the surgeon's ability to optimize outcomes in terms of facial symmetry. ARN also has significant educational value, allowing surgeons and trainees to better understand neuroanatomy and the surgical challenges associated with CFD.

The accuracy achieved in this case, with a deviation of 2 mm, compares favorably to both freehand techniques and the results reported by Liu et al, 11 who found an accuracy of 1.44 mm using similar ARN-assisted methods. The slight differences in accuracy in this study could be attributed to the anatomical location of the dysplastic bone, which was in the parietal region. This region is characterized by the intersection of multiple cranial bones, making reshaping more complex compared with surgical resections performed anteriorly or in more accessible areas. 16 This factor highlights the need for further refinement in ARN technology, particularly when operating in regions with complex osseous intersections.

## Limitations

Despite the positive outcomes observed in this case, there are limitations to the use of ARN in CFD surgery. The accuracy of ARN systems can be influenced by factors such as surgical site complexity and occlusion during the registration process, as well as anatomical variability and the presence of tumor-related deformities. Future studies should focus on improving ARN accuracy across different anatomical regions and on developing more sophisticated algorithms for real-time patient anatomy registration. Further research is also needed to assess its long-term impact on clinical outcomes and complication rates. Prospective studies with larger sample sizes will be essential to validate the findings of this case and to determine the broader applicability of ARN in the treatment of CFD and other complex craniofacial conditions.

## Conclusion

ARN technology is a valuable tool for enhancing the precision and safety of CFD surgery, offering the potential to improve both functional and aesthetic outcomes. By allowing real-time visualization of critical structures, ARN assists in navigating complex anatomy and achieving better facial symmetry with minimal damage to neurovascular structures. As ARN systems continue to evolve, their role in craniofacial surgery is likely to expand, benefiting both clinical practice and surgical education. This case report proposes additional controls in the treatment of CFD, particularly by incorporating the visualization of critical neurovascular structures, which further enhances surgical safety and outcomes.
